# Chronic kidney disease and transvenous cardiac implantable electronic device infection—is there an impact on healthcare utilization, costs, disease progression, and mortality?

**DOI:** 10.1093/europace/euae169

**Published:** 2024-06-19

**Authors:** David J Wright, María Emilce Trucco, Jiani Zhou, Claudia Wolff, Reece Holbrook, Jamie Margetta, Mikhael F El-Chami

**Affiliations:** Cardiology Division, Liverpool Heart and Chest Hospital, Liverpool, UK; Arrhythmia Section, Cardiology Department, Hospital Universitari Doctor Josep Trueta and Institut d'Investigació Biomèdica de Girona (IDIBGI), Girona, Spain; Cardiac Rhythm Management, Medtronic plc, 8200 Coral Sea Street, MVC71 Mounds View, MN 55112, USA; Medtronic International Trading Sàrl, Tolochenaz, Switzerland; Cardiac Rhythm Management, Medtronic plc, 8200 Coral Sea Street, MVC71 Mounds View, MN 55112, USA; Cardiac Rhythm Management, Medtronic plc, 8200 Coral Sea Street, MVC71 Mounds View, MN 55112, USA; Department of Medicine, Division of Cardiology, Emory University Hospital, Atlanta, GA, USA

**Keywords:** CIED infection, Chronic kidney disease, Healthcare resources, Costs, Disease progression, Mortality

## Abstract

**Aims:**

Cardiac implantable electronic device (CIED) infections are a burden to hospitals and costly for healthcare systems. Chronic kidney disease (CKD) increases the risk of CIED infections, but its differential impact on healthcare utilization, costs, and outcomes is not known.

**Methods and results:**

This retrospective analysis used de-identified Medicare Fee-for-Service claims to identify patients implanted with a CIED from July 2016 to December 2020. Outcomes were defined as hospital days and costs within 12 months post-implant, post-infection CKD progression, and mortality. Generalized linear models were used to calculate results by CKD and infection status while controlling for other comorbidities, with differences between cohorts representing the incremental effect associated with CKD. A total of 584 543 patients had a CIED implant, of which 26% had CKD and 1.4% had a device infection. The average total days in hospital for infected patients was 23.5 days with CKD vs. 14.5 days (*P* < 0.001) without. The average cost of infection was $121 756 with CKD vs. $55 366 without (*P* < 0.001), leading to an incremental cost associated with CKD of $66 390. Infected patients with CKD were more likely to have septicaemia or severe sepsis than those without CKD (11.0 vs. 4.6%, *P* < 0.001). After infection, CKD patients were more likely to experience CKD progression (hazard ratio 1.26, *P* < 0.001) and mortality (hazard ratio 1.89, *P* < 0.001).

**Conclusion:**

Cardiac implantable electronic device infection in patients with CKD was associated with more healthcare utilization, higher cost, greater disease progression, and greater mortality compared to patients without CKD.

## Graphical Abstract

**Graphical Abstract:**
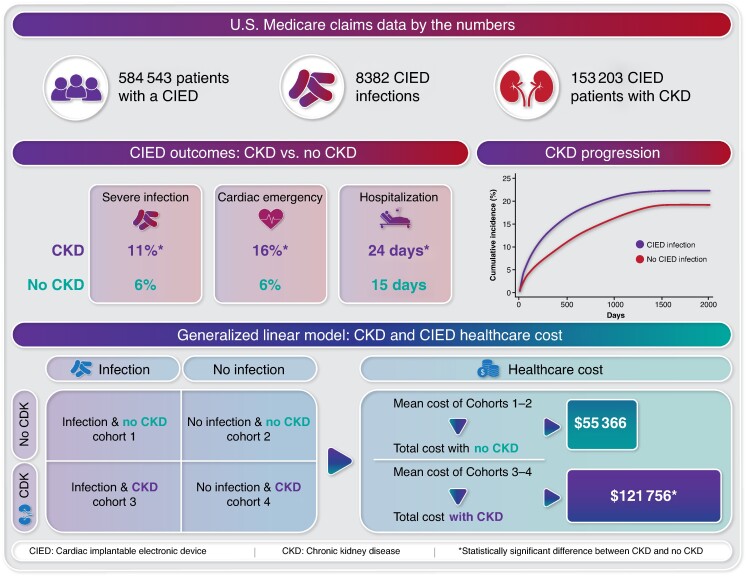

**What’s new?**

- First comparison of healthcare utilization, costs, disease progression, and mortality associated with cardiac implantable electronic device (CIED) infection in patients with chronic kidney disease (CKD) to patients without CKD.
- The results demonstrate that CIED infections are more severe for patients with CKD than for patients without CKD.
- Patients with an infection were more likely to progress to a higher state of CKD and experienced a higher mortality than CKD patients without infection.
- Infections are twice as expensive to treat for a patient with CKD.
- Strategies to reduce infection probability in patients with moderate-to-severe chronic kidney disease could lead to significant clinical and economic benefits.

## Introduction

Cardiac implantable electronic devices (CIEDs) are an important treatment for cardiac arrhythmias to reduce symptoms, improve quality of life, and increase survival in selected patients.^1–5^ Although CIEDs are an effective and safe therapy, complications still occur. Cardiac device infections are one of the most serious CIED complications. Approximately 1–4% of CIED patients experience a cardiac device infection depending on the type of device and procedure.^6–8^

Furthermore, certain comorbidities such as diabetes mellitus, chronic kidney disease (CKD), and chronic obstructive pulmonary disease increase the risk of CIED infection.^9^ Patients with CKD are an important high-risk cohort with several studies showing a significantly higher risk of CIED infection. For patients with renal insufficiency, the risk of infection was two to four times higher and nine times higher for patients with end-stage renal disease.^9–12^

Cardiac implantable electronic device infections cause a significant increase in healthcare utilization and costs^13–18^ as well as a temporary reduction in quality of life.^14^ More importantly, CIED infections are associated with a significant increase in mortality that is influenced by the presence of multiple comorbidities.^13,14,18–23^ Patients with CKD have lower life expectancy compared to patients without this chronic condition.^24^ A CIED infection could amplify the difference in this patient population.

In this study, we compare the severity, healthcare utilization, and costs of an infection in CKD patients to patients without this chronic condition. We also analyse the progression of CKD and mortality in CKD patients with and without CIED infection. Finally, we evaluate how stage of CKD impacts outcomes of interest.

## Methods

### Cohort identification

This is a retrospective study that uses the Medicare Limited Data Set standard analytic files, a large administrative claims data set of Medicare Fee-for-Service (FFS) beneficiaries from the USA. The Medicare data cover people aged 65 years or older, younger people with disabilities, and people with end-stage renal disease. This data set follows patients longitudinally and provides a large sample of patients implanted with CIEDs. Medicare data include the inpatient and outpatient settings, although they do not include claims from skilled nursing or long-term care facilities. This study was designed to be consistent with the Strengthening the Reporting of Observational Studies in Epidemiology guidelines for retrospective studies.^25^

This study is exempted from Institutional Review Board review based on its de-identified patient status and the obtained exemption status under the Health Insurance Portability and Accountability Act with a full waiver of authorization for use.

**Figure 1:**
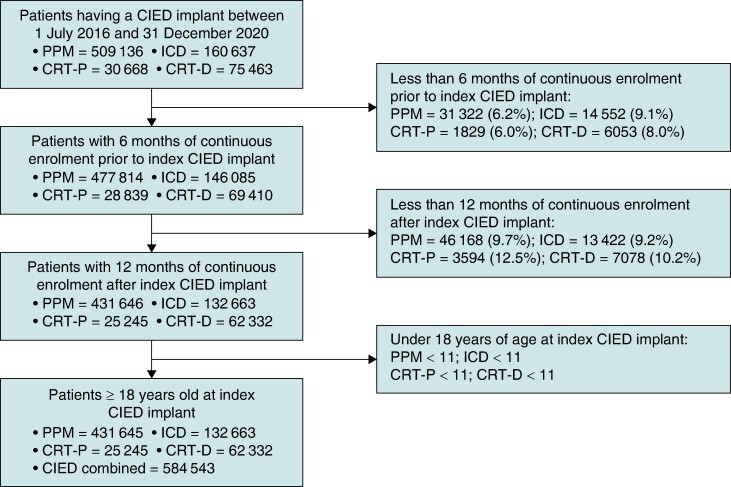
Attrition diagram of study cohort. CIED, cardiac implantable electronic device; CRT-D, cardiac resynchronization therapy defibrillator; CRT-P, cardiac resynchronization therapy pacemaker; ICD, implantable cardioverter-defibrillator; PPM, permanent pacemaker.

Patients were included if they received a CIED implant (where implant could signify *de novo*, replacement, or upgrade device) between 1 July 2016 and 31 December 2020. Cardiac implantable electronic device implant years were limited to 2016–2020 to ensure availability of 6 months’ continuous enrolment prior to CIED implant and adequate follow-up post–device implant during the ICD-10-CM coding era (the transition to ICD-10-CM from ICD-9-CM occurred on 1 October 2015). Analyses were conducted on distinct device type cohorts: implantable cardioverter-defibrillator (ICDs), permanent pacemakers (PPMs), and cardiac resynchronization therapy (CRTs), as well as the total CIED cohort analysis. Patients were allowed to contribute to multiple device type cohorts if unique index dates were obtained for separate device types. The overall CIED cohort was restricted to the minimum index date and associated device per patient. Patients were required to be above the age of 18 at implant and be enrolled in both Medicare Part A (inpatient coverage) and Part B (outpatient coverage) to be included in the analysis (*Figure 1*).

### Follow-up

This study had two distinct follow-up periods. The first follow-up period was 12 months from the date of CIED implant and is referred to as the infection window. The length of the infection window was chosen to balance the availability of follow-up data and a specified time period for cost and infection rate characterization. The second follow-up period includes all available follow-up data and is referred to as total follow-up period (until December 2021).

#### Patient selection

The first observed CIED implant for each patient during the study period was selected to establish the study cohort; the date associated with CIED implant was defined as the index date. The appropriate *International Classification of Disease-10th Revision Procedure Codes and Current Procedural Terminology (CPT)* were used to identify patients’ baseline comorbidities (see Supplementary material online, *Table S1*). Those with at least one CKD code in the inpatient or outpatient setting during the 6 months prior to their CIED implant date were assigned to the CKD group, while patients without any CKD codes within the 6 months before the implant date were assigned to the non-CKD group (see Supplementary material online, *Table S2*).

Infection classification was based on patients having at least one claim for a CIED infection, identified by the ICD-10-CM diagnosis code T82.7XXA, during the infection window.

**Figure 2:**
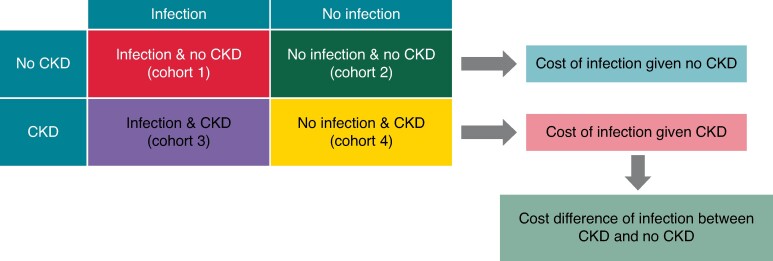
Cohort construction for cost difference analysis. CKD, chronic kidney disease.

Patients were further classified into four cohorts as illustrated in *Figure 2*: Cohort 1, patients who experienced an infection during follow-up with no CKD at baseline; Cohort 2, patients who did not experience an infection during follow-up with no CKD at baseline; Cohort 3, patients who experienced an infection during follow-up who have CKD at baseline; and Cohort 4, patients who did not experience an infection during follow-up who have CKD at baseline.

### Outcome measures

This study assessed various outcomes associated with the presence of CKD and infections among CIED patients. Inpatient healthcare utilization (HCU) includes the number of inpatient admissions and length of stay (LOS). Total healthcare costs were calculated by combining both inpatient and outpatient costs during the infection window. The severity of device infections was assessed by examining relevant diagnosis-related group (DRG) codes. Specifically, DRG code 291 was used for evaluating heart failure (HF) and cardiogenic shock with major complication or comorbidity (MCC), DRG code 871 was used for septicaemia or severe sepsis without mechanical ventilation > 96 h with MCC, and DRG code 003 was used to identify extracorporeal membrane oxygenation (ECMO) use, and lead extractions were identified (CPT, 33243, 33244; ICD-10-PCS, 02PA3MZ, 02PA0MZ) to further understand the impact of infections on patients with CKD.

Additional outcome variables of the study were CKD progression and mortality. Chronic kidney disease progression was defined as the advancement to a higher stage of CKD. Both CKD progression and mortality were evaluated for the total follow-up period.

### Statistical analysis

Baseline characteristics of patients were compared between groups using appropriate statistical tests—*χ*^2^ tests (or Fisher’s exact tests) for categorical variables and paired sample *t*-tests, Student’s tests, or Mann–Whitney *U* tests for continuous variables.

A generalized linear model was utilized to calculate healthcare costs of patients with and without CKD and CIED infection. The mean cost was estimated for all four cohorts. The differences in mean costs between Cohorts 1 and 2 represent the total infection cost without CKD, and differences in mean costs between Cohorts 3 and 4 represent total infection cost with CKD (*Figure 2*). The simple differences of costs without CKD and costs with CKD highlight the incremental cost of infected patients with CKD. The same model and methods were applied to HCU to calculate the incremental HCU of infected patients with CKD. A Cox proportional hazards function was utilized to examine differences in CKD progression and mortality after infection between patients with CKD and those without CKD.

The covariates in the adjusted model were selected based on factors shown to be associated with healthcare costs and infection risk.^9,26,27^ The covariates were history of dialysis, prior CIED procedure (non-implant), age, race, geographic region, atrial fibrillation, diabetes, HF, hypertension, and immunocompromised condition. Numerical results are presented as mean [standard deviation (SD)], median [interquartile range (IQR)], or number (percentage). All statistical tests were two-sided, and significance was set at *P* < 0.05. Statistical computations were carried out using SAS Studio (SAS Institute Inc., Cary, NC).

## Results

### Baseline characteristics

Between 1 July 2016 and 31 December 2020, 584 453 patients in the US Medicare claims database underwent a CIED implantation and were included in the analysis. The average age was 76.5 ± 9.0 years, 42.6% were female, and 88.1% White. A total of 153 203 patients (26.2%) had a code for CKD in baseline.

A total of 8382 patients (1.4%) experienced at least one CIED infection during the defined infection window. When stratifying infection by device type, the largest infection rate of 2.3% was seen in the CRT cohort, followed by 1.9% in the ICD cohort and 1.2% in the PPM cohort (*P* < 0.001). Of the infected patients, 40% (*n* = 3322) had a history of CKD at baseline. Among the non-infected patients, 26% (*n* = 149 881) had a history of CKD at baseline (*Table 1*).

**Table 1:** Baseline characteristics of patients with CIED between July 2016 and December 2020

	Total	Infected with no CKD (ref.)	Infected with CKD	Non-infected with no CKD (ref.)	Non-infected with CKD
*n*	584 543	5060	3322	426 280	149 881
Device type^a^					
PPM	416 523	2985	1769	311 331	100 438
ICD	84 622	1024	744	59 022	23 832
CRT	83 398	1051	809	55 927	25 611
Device infection in 12 months	8382 (1.4%)				
Baseline CKD	153 203 (26.2%)				
CKD stage at baseline					
Stage 1	988 (0.2%)		11 (0.3%)		977 (0.7%)
Stage 2	9206 (1.6%)		159 (4.8%)		9047 (6.0%)
Stage 3	75 221 (12.9%)		1282 (38.6%)		73 939 (49.3%)
Stage 4	13 055 (2.2%)		264 (8.0%)		12 791 (8.5%)
Stage 5	857 (0.2%)		29 (0.9%)		828 (0.6%)
End-stage renal disease	12 107 (2.1%)		810 (24.4%)		11 297 (7.5%)
Unspecified	41 769 (7.2%)		767 (23.1%)		41 002 (27.4%)
Age (mean/SD)	76.5 (9.0)	73.6 (10.9)	72.5 (11.2)***	76.3 (8.8)	77.2 (9.3)***
Age ≥ 65	546 827 (93.6%)	4347 (85.9%)	2687 (80.9%)***	400 930 (94.1%)	138 863 (92.7%)***
Female, sex	249 048 (42.6%)	1853 (36.6%)	1102 (33.2%)**	186 159 (43.7%)	59 934 (40.0%)***
White, race/ethnicity	514 683 (88.1%)	4533 (89.6%)	2600 (78.3%)***	381 847 (89.6%)	125 703 (83.9%)***
Geographic region					
Northeast	138 692 (23.7%)	1120 (22.1%)	736 (22.2%)	101 324 (23.8%)	35 512 (23.7%)
Midwest	102 434 (17.5%)	875 (17.3%)	572 (17.2%)	74 192 (17.4%)	26 795 (17.9%)***
South	218 412 (37.4%)	1982 (39.2%)	1348 (40.6%)	159 840 (37.5%)	55 242 (36.9%)***
West	71 255 (12.2%)	632 (12.5%)	358 (10.8%)*	52 538 (12.3%)	17 727 (11.8%)***
Unknown	53 750 (9.2%)	451 (8.9%)	308 (9.3%)	38 386 (9.0%)	14 605 (9.7%)***
Comorbidities					
Atrial fibrillation	233 113 (39.9%)	2278 (45.0%)	1872 (56.4%)***	155 501 (36.5%)	73 462 (49.0%)***
Diabetes	179 917 (30.8%)	1439 (28.4%)	1949 (58.7%)***	102 024 (23.9%)	74 505 (49.7%)***
HF	181 258 (31.0%)	1963 (38.8%)	2165 (65.2%)***	104 565 (24.5%)	72 565 (48.4%)***
Hypertension	421 415 (72.1%)	3663 (72.4%)	3077 (92.6%)***	280 824 (65.9%)	133 851 (89.3%)***
Immunocompromised condition	10 999 (1.9%)	103 (2.0%)	104 (3.1%)**	6849 (1.6%)	3943 (2.6%)***
Dialysis in 6 months prior to index implant	937 (0.2%)	3 (0.06%)	50 (1.5%)***	105 (0.02%)	779 (0.5%)***
CIED procedures in 6 months prior to index implant	3076 (0.5%)	498 (9.8%)	403 (12.1%)***	1263 (0.3%)	912 (0.6%)***

CIED, cardiac implantable electronic device; CKD, chronic kidney disease.

^a^Patients were allowed to contribute to multiple device type cohorts if unique index dates were obtained for separate devices. The overall CIED cohort was restricted to the minimum index date and associated device.

*P < 0.05; **P < 0.01; ***P < 0.001

Among all CIED patients, 40% had atrial fibrillation, 31% had diabetes, 31% had HF, 72% had hypertension, and 2% had an immunocompromised condition. A small fraction underwent dialysis (0.2%) or CIED procedures (0.5%) in the 6 months preceding implantation. Patients with CKD, in comparison to those without CKD, tended to be younger, more likely to be male and non-White, and resided in the western region. In addition, they were more likely to have comorbidities, undergo dialysis, and to have had other CIED procedures prior to implantation (*Table 1*).

### Healthcare resource utilization

The presence of infections was associated with an average of 1.7 [95% confidence interval (CI), 1.6–1.8] additional hospitalizations for patients with CKD and an average of 1.4 (95% CI, 1.3–1.4) additional hospitalizations for patients without CKD, resulting in an additional 0.4 hospitalizations with CKD (95% CI, 0.3–0.5; *Table 2*). The average days in hospital for infected patients with CKD were 23.5 days (95% CI, 21.5–25.4) vs. 14.5 days (95% CI, 13.6–15.4) for infected patients without CKD resulting in an additional 9 days in hospital for patients with CKD (95% CI, 6.8–11.1).

**Table 2:** Healthcare resource utilization in patients with CIED

	Number of inpatient admissions			LOS (day)		
	Mean	95% CI	*P* value	Mean	95% CI	*P*-value
No CKD	1.4	(1.3, 1.4)	<0.001	14.5	(13.6, 15.4)	<0.001
CKD	1.7	(1.6, 1.8)	<0.001	23.5	(21.5, 25.4)	<0.001
Difference	0.4	(0.3, 0.5)	<0.001	9.0	(6.8, 11.1)	<0.001
*n*	584 534			584 534		

CI, confidential interval; CIED, cardiac implantable electronic device; CKD, chronic kidney disease.

### Healthcare costs

**Figure 3:**
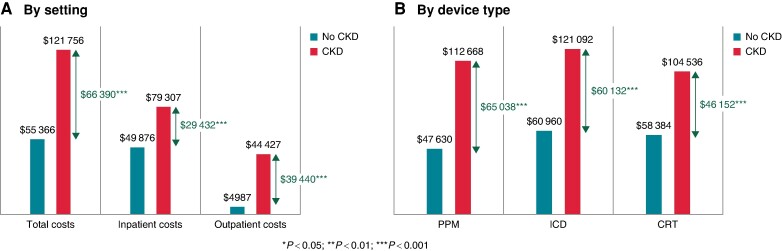
Healthcare costs in patients with CIED infection. CIED, cardiac implantable electronic device; CRT, cardiac resynchronization therapy; ICD, implantable cardioverter-defibrillator; PPM, permanent pacemaker, CKD, chronic kidney disease.

Patients with CKD experienced an average total cost of device-related infection of $121 756 (95% CI, $110 956–$132 556), compared to patients without CKD who experienced an average total cost of device-related infection of $55 366 (95% CI, $51 180–$59 551). The difference in total costs of device-related infection between these cohorts was statistically significant at $66 391 (95% CI, $54 818–$77 963; *Figure 3*). There were increased costs of infection in CKD patients for all device types (*Figure 3*) and at all stages of CKD, being statistically significant from Stage 3 forward (see Supplementary material online, *Figure S1*).

The average inpatient costs for a CKD patient with an infection was $79 307 (95% CI, $45 044–$54 707) vs. $49 876 (95% CI, $45 044–$54 707) for a patient with an infection and no CKD. The incremental inpatient costs of an infection in CKD as compared to no CKD was $29 432 (95% CI, $18 251–$40 612). Patients with CKD experienced an average outpatient cost of device-related infection of $44 427 (95% CI, $39 857–$48 998), compared to $4987 (95% CI, $4001–$5974) for patients with an infection without CKD. The difference in outpatient costs in these cohorts was $39 440 (95% CI, $34 771–$44 109).

### Severity of infections

**Figure 4:**
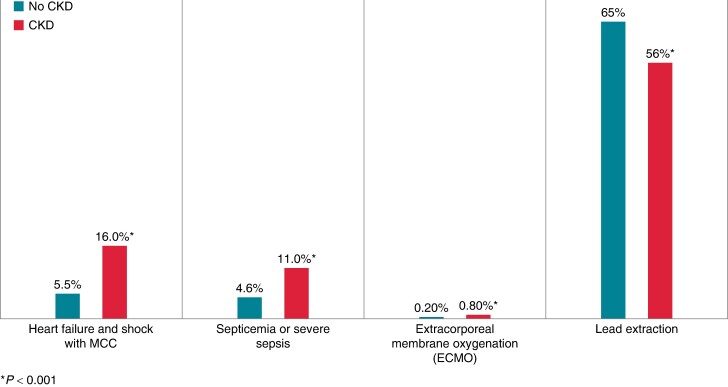
Measures of severity of CIED infection by CKD status. CIED, cardiac implantable electronic device; CKD, chronic kidney disease.

Among patients affected by device infections, those with CKD were more likely to experience HF and cardiogenic shock than those without CKD (16.0 vs. 5.5%; *P* < 0.001). Additionally, the incidence of septicaemia or severe sepsis was markedly elevated in patients with CKD compared to those without CKD (11.0 vs. 4.6%; *P* < 0.001). Patients with CKD and infection needed ECMO more often than patients with infections and no CKD (0.8% for patients with CKD vs. 0.2% for patients without CKD, *P* < 0.001). Conversely, patients with CKD had less lead extraction procedures than patients without CKD (56 vs. 65%; *P* < 0.001; *Figure 4*).

### Chronic kidney disease progression and mortality

**Figure 5:**
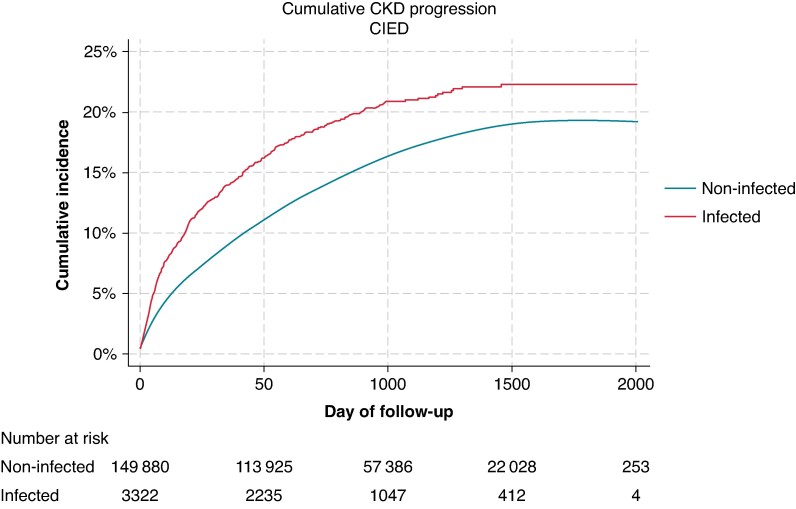
Cumulative incidence of CKD progression in patients with CIED by infection status. CIED, cardiac implantable electronic device; CKD, chronic kidney disease.

**Figure 6:**
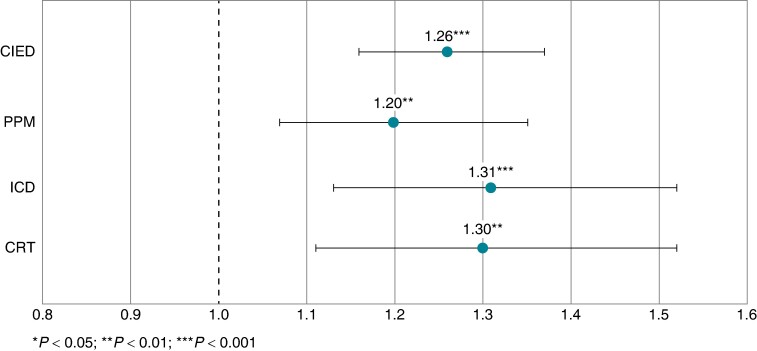
Chronic kidney disease progression in patients with CIED by device type. CRT-D, cardiac resynchronization therapy defibrillator; CRT-P, cardiac resynchronization therapy pacemaker; ICD, implantable cardioverter-defibrillator; PPM, permanent pacemaker; CKD, chronic kidney disease.

**Figure 7:**
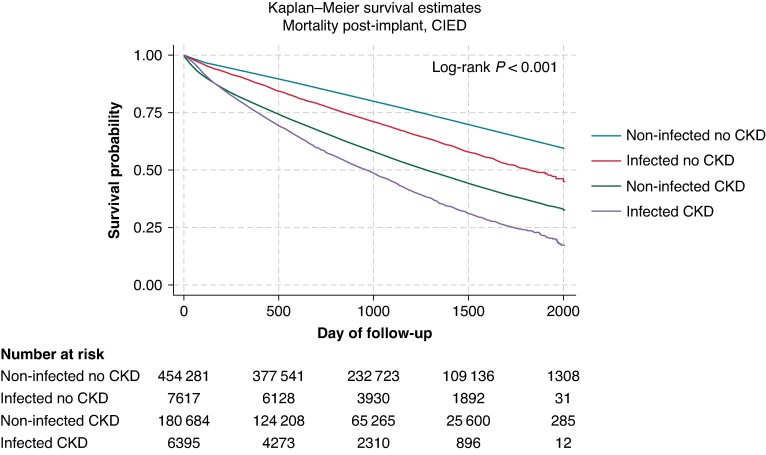
Mortality by CKD and infection status. CIED, cardiac implantable electronic device; CKD, chronic kidney disease.

Compared to those without infections, patients who were infected post-implant demonstrated an increased risk of CKD progression throughout the post-implant follow-up period (*Figure 5*). After adjustments for demographic factors and baseline comorbidities, infected patients were 1.26 times more likely to experience CKD progression compared to those without infections (95% CI, 1.16–1.37). These differences in the risk of CKD progression remain consistent across device types (*Figure 6*). After infection, CKD patients were 1.89 times more likely to experience mortality than non-CKD patients (95% CI, 1.79–2.00; *Figure 7*).

## Discussion

This study characterizes the impact of CKD on healthcare utilization, costs, disease progression, and mortality of CIED infections. Chronic kidney disease was associated with a significant increase in hospitalizations, total days spent in the hospital, and annual healthcare spending. Chronic kidney disease patients were more likely to have increased severity of infection as characterized by increased post-infection incidence of sepsis, hospitalization for HF and cardiogenic shock, and use of ECMO. Patients with CKD that had a CIED infection were more likely to experience CKD progression or mortality than those that did not have an infection.

This study is based on a contemporaneous Medicare population and provides valuable data beyond what is captured in clinical trials and post-approval registries, as clinical studies of device infections generally do not provide a large-enough sample size to sub-stratify patients with infections into CKD and non-CKD subgroups. We found differences between adjusted and unadjusted results that illustrate the relevance of addressing significantly different patient characteristics between the four study cohorts via appropriate design and statistical techniques.

The presence of CKD may be associated with higher burden of infection due to the accumulation of uraemic toxins that lead to dysfunction of the immune system in the presence of multiple comorbidities.^28,29^ This might explain not only the higher infection rate in this group of patients but also the increased severity of infection. Progression of CKD is likely to have an adverse impact on patients’ quality of life and survival.^30–32^ The association of CIED infections with increased mortality has been shown in various studies.^13,14,18–23^ However, for patients with CKD, the effect appears to be more dramatic, perhaps because their life expectancy is already significantly shorter due to the chronic condition and possibly due to the more rapid progression of CKD in the setting of CIED infection. Additionally, the reduced use of lead extraction in CKD patients likely limits the effectiveness of the post-infection treatment. This observation may reflect that the extraction procedure is considered riskier as CKD patients are sicker, may have a central catheter due to haemodialysis, and may have a higher degree of lead calcification making the procedure challenging and increasing risk of vascular damage. Chronic kidney disease has been associated with worse outcomes and higher mortality after lead extraction procedures.^33,34^

While the difference in costs is significantly increased in the presence of CKD, this difference does not seem to be fully explained by the higher number of hospital admissions and the longer length of stay. There may be additional care related to the treatment of CKD that adds to the difference in costs, such as the increased incidence of sepsis and use of ECMO. Additionally, treatment costs rise exponentially by stage of CKD, and thus, a CIED infection that led to a worsening of CKD might translate into permanently higher healthcare costs for the life of the patient.^35^

Given the significant impact of a CIED infection on a CKD patient and the high costs of an infection in these patients, prevention of a CIED infection is very important. Strategies to minimize the risk of CIED infections include the use of commercially available antibacterial envelopes. The randomized WRAP-IT trial reported a 40% reduction in major CIED infections and 61% reduction in pocket infections within 12 months of the procedure with the use of an antibacterial envelope.^36^ The efficacy of the envelope has since been corroborated in other settings.^37–39^ The use of the antibacterial envelope in the WRAP-IT population and in high-risk subgroups and procedures was recommended in the 2019 European Heart Rhythm Association (EHRA) International Consensus document and in the 2021 ESC Pacing Guidelines in Pacemaker re-interventions (Class IIb).^18,40^

For CKD patients in need of a pacemaker, a leadless pacemaker could be an alternative than conventional pacemakers due to the very low risk of infection. Over 200 000 leadless pacemakers have been implanted since the first such device was made commercially available in 2015, and very few infections have been reported.^41–44^ The lower risk of infection is likely related to smaller device size, the elimination of the pacemaker pocket and leads, the parylene coating, the turbulent haemodynamic environment, and the reduced handling of the leadless pacemaker.^45,46^ Additional leadless pacemakers became commercially available in 2022, and the clinical studies have reported no infection for both devices.^47,48^ Leadless pacemakers are recommended in the 2021 ESC Pacing Guidelines in patients with a high risk of pocket infection (Class IIa) as well as in an EHRA position paper and in Austrian and British consensus papers on leadless pacing.^18,49–51^ The EHRA international consensus document on how to prevent, diagnose, and treat infections recommends leadless pacing as an excellent alternative in selected patients at high risk of infection or with previous infection.^18^ Similarly, CKD patients who are candidates for an ICD might benefit, when appropriate, from an extravascular ICD or a subcutaneous ICD to minimize the risk of systemic infections more commonly seen with transvenous leads.^52,53^

The results of this study confirm the hypothesis that CIED infections are more burdensome across all measures for patients with CKD. There has been a focus on the potential complications of CIEDs in patients with advanced CKD, but this analysis demonstrates that there can be clinical and economic benefits in taking measures to avoid infections even in earlier stages of CKD.

There are several limitations inherent to this kind of nonrandomized observational study. First, claims data provide lesser context than a conventional clinical trial. It was impossible to determine if multiple healthcare utilizations for infection were one or multiple infections. This was mitigated by analysis methods that measured annual costs and utilization and time to first event survival for mortality and disease progression.

Patients with renal disease often have other severe comorbidities or conditions that could have an impact on the severity, mortality, healthcare utilization, and costs of an infection. In this analysis, we control for several comorbidities that have been shown to be common in patients with CKD and to impact healthcare costs (atrial fibrillation, diabetes, HF, hypertension, and immunocompromised conditions). We also control for prior CIED procedure and history of dialysis as these characteristics increase the risk of infection. However, there could be unobservable differences that we cannot account for in the analysis that increase the risk of an infection and have an impact on the outcome variables.

The study was restricted to CIED infections that occurred within the first 12 months following device implantation due to the available data. Cardiac implantable electronic device infections that might have occurred during later years could have had an impact on the mortality and CKD disease progression outcomes. The study was also limited in the specific history of patients prior to the 6-month baseline period of the study and may underestimate the prevalence of some baseline covariates such as prior CIED procedures. While the Cox proportional hazard model did censor patients at time of death, the model did not control for death as a competing risk factor that could have potential effects on overall associations.

This analysis was limited to the Medicare FFS population, which primarily consists of patients 65 years and older and those with certain disabilities or end-stage renal disease. Thus, the results may not be generalizable to populations outside the US Medicare FFS population, particularly those who are younger, as younger age has been associated with increased risk for infection.

## Conclusion

In a large US claims data set, CKD was associated with more and longer hospitalizations and costs that were more than twice as high. Chronic kidney disease was associated with more severe CIED infections as measured by sepsis, HF hospitalizations, and ECMO use. In addition, patients with CKD were associated with a higher likelihood to progress to a worse stage of CKD and lower survival after an infection.

## Supplementary Material

euae169_Supplementary_Data

## Data Availability

The claims database that supports the findings of this study is owned by the Centers for Medicare and Medicaid Services and was used under licence for the current study, so restrictions apply and the data are not publicly available.
