# Supplementary material for: Chronic kidney disease and transvenous cardiac implantable electronic device infection—is there an impact on healthcare utilization, costs, disease progression, and mortality?
Source: Europace. 2024 Jun 19;26(7):euae169. doi: 10.1093/europace/euae169 (PMC11223657; doi:10.1093/europace/euae169)

**Supplementary Data**

**Table S1 Baseline Comorbidity Codes**

**Dialysis codes:**

| ICD PSC | 10 | 5A1D70Z,5A1D80Z,5A1D90Z | Dialysis procedure |  |
| --- | --- | --- | --- | --- |
| CPT |  | 90935,90937 | Dialysis procedure |  |

| **IMMUNOCOMPROMISED CODES:**  **ICD-10-CM** | **Description** |
| --- | --- |
| **Human immunodeficiency virus [HIV] disease /AIDS** |  |
| B20 | Human immunodeficiency virus [HIV] disease |
| Z21 | Asymptomatic human immunodeficiency virus [HIV] infection status |
|  |  |
| **mononucleosis** |  |
| B27.00 | Gammaherpesviral mononucleosis without complication |
| B27.01 | Gammaherpesviral mononucleosis with polyneuropathy |
| B27.02 | Gammaherpesviral mononucleosis with meningitis |
| B27.09 | Gammaherpesviral mononucleosis with other complications |
| B27.10 | Cytomegaloviral mononucleosis without complications |
| B27.11 | Cytomegaloviral mononucleosis with polyneuropathy |
| B27.12 | Cytomegaloviral mononucleosis with meningitis |
| B27.19 | Cytomegaloviral mononucleosis with other complication |
| B27.80 | Other infectious mononucleosis without complication |
| B27.81 | Other infectious mononucleosis with polyneuropathy |
| B27.82 | Other infectious mononucleosis with meningitis |
| B27.89 | Other infectious mononucleosis with other complication |
| B27.90 | Infectious mononucleosis, unspecified without complication |
| B27.91 | Infectious mononucleosis, unspecified with polyneuropathy |
| B27.92 | Infectious mononucleosis, unspecified with meningitis |
| B27.99 | Infectious mononucleosis, unspecified with other complication |
| **Malignant Neoplasm of Lymphatic and Hematopoietic Tissue** |  |
| C90 | Plasma cell leukemia |
| C91 | Lymphoid leukemia |
| C92 | Myeloid leukemia |
| C93 | Monocytic leukemia |
| C94 | Other leukemias of specified cell type |
| C95 | Leukemia of unspecified cell type |
| C81 | Hodgkin lymphoma |
| C82 | Follicular lymphoma |
| C83 | Non-follicular lymphoma |
| C85 | Other specified and unspecified types of non-Hodgkin lymphoma |
| C86 | Other specified types of T/NK-cell lymphoma |

**Prior Procedures:**

|  | **CPT code** |  | **ICD-10-CM** |
| --- | --- | --- | --- |
| **PPM** |  | | |
| Initial System Implant | 33206, 33207, 33208 | | Generator: 0JH604Z or 0JH605Z or 0JH606Z |
|  |  |  | Leadless: 02HK3NZ |
|  |  |  | Leads: 02H63JZ and/or 02HK3JZ |
| Generator Implant Only | 33212, 33213 | | 0JH604Z or 0JH605Z or 0JH606Z |
|  |  |  | Leadless: 02HK3NZ |
| Replacement | 33227, 33228 | | 0JH604Z or 0JH605Z or 0JH606Z and 0JPT0PZ |
| Upgrade | 33214 | | 0JPT0PZ and 0JH606Z and 02H63JZ or 02HK3JZ |
| **ICD** | | | |
| Initial System Implant | 33249 | | 0JH608Z and 02H63KZ and/or 02HK3KZ |
| Generator Implant Only | 33230, 33240 | | 0JH608Z |
| Replacement | 33262, 33263 | | 0JPT0PZ and 0JH608Z |
| Upgrade | 33233 + 33249 | | 0JPT0PZ and 0JH608Z and 02H63KZ and/or 02HK3KZ |
| **CRT-P** | | | |
| Initial System Implant | 33206 + 33225, 33207 + 33225, 33208 + 33225 | | 0JH607Z and 02H63JZ and/or 02HK3JZ and 02H43JZ or 02HL3JZ |
| Generator Implant Only | 33212, 33213 | | 0JH607Z |
| Replacement | 33221, 33229 | | 0JH607Z and 0JPT0PZ |
| Upgrade | 33214 + 33225 | | 0JPT0PZ and |
|  |  |  | 02H63JZ or 02HK3JZ and 02H43JZ or 02HL3JZ |
| **CRT-D** | | | |
| Initial implant system | 33249 + 33225 | | 0JH609Z and 02H63KZ and/or 02HK3KZ and 02H43KZ or 02HL3KZ |
| Generator Implant Only | 33231 | | 0JH609Z |
| Replacement | 33264 | | 0JPT0PZ and 0JH609Z |
| Upgrade | 33233 + 33249 + 33225 | | 0JPT0PZ and 0JH609Z and 02H63KZ and/or 02HK3KZ and 02H43KZ or 02HL3KZ |
| **Codes shared by CIED** | | | |
| Pocket Revision | 33222, 33223 | | 0JWT0PZ (this is for revision |
|  |  |  | only not relocation) |
| Lead Revision | 33215 | | 02WA3MZ |
| Lead Only | PPM/ICD: 33216, 33217, 33271 | | 02H63JZ, 02HK3JZ, 02H43JZ, 02HL3JZ, 02H63KZ, 0JH63FZ, 02HK3KZ, 02H43KZ or 02HL3KZ (any of these codes would represent insertion of lead or leads) |
|  | CRT: 33224, 33225 | |  |

**Table S2. CKD codes**

| ICD DX | 10 | N18.1 | Chronic kidney disease, stage 1 | Kidney Disease |
| --- | --- | --- | --- | --- |
| ICD DX | 10 | N18.2 | Chronic kidney disease, stage 2 (mild) | Kidney Disease |
| ICD DX | 10 | N18.30 | Chronic kidney disease, stage 3 unspecified | Kidney Disease |
| ICD DX | 10 | N18.31 | Chronic kidney disease, stage 3a | Kidney Disease |
| ICD DX | 10 | N18.32 | Chronic kidney disease, stage 3b | Kidney Disease |
| ICD DX | 10 | N18.4 | Chronic kidney disease, stage 4 (severe) | Kidney Disease |
| ICD DX | 10 | N18.5 | Chronic kidney disease, stage 5 | Kidney Disease |
| ICD DX | 10 | N18.6 | End stage renal disease | Kidney Disease |
| ICD DX | 10 | N18.9 | Chronic kidney disease, unspecified | Kidney Disease |

**Figure S1: Difference in total healthcare costs in patients with CIED, by device type and CKD stage**


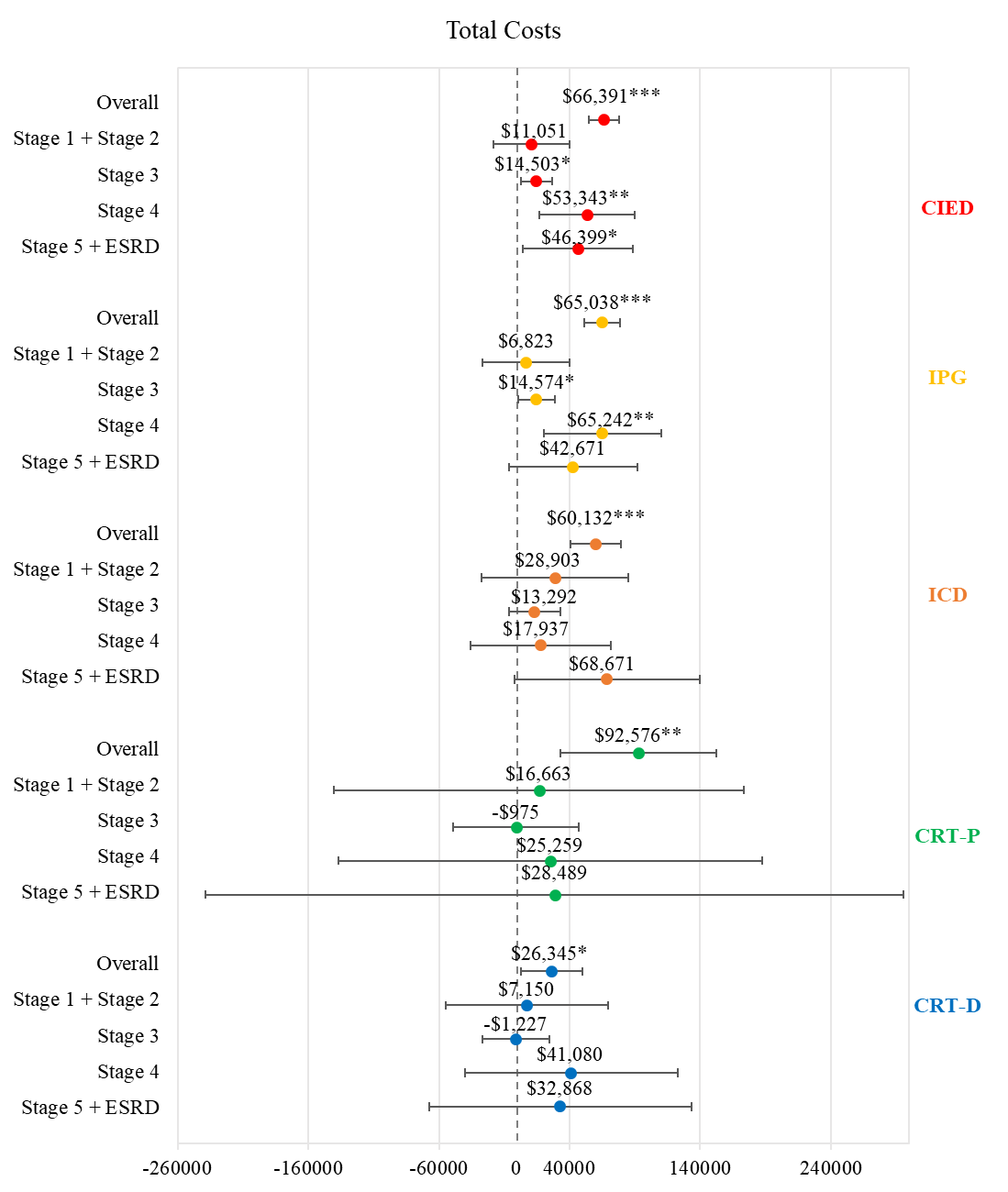

Supplement: euae169_Supplementary_Data [file euae169_supplementary_data.docx]
